# Numerical Study on Broadband Antireflection of Moth-Eye Nanostructured Polymer Film with Flexible Polyethylene Terephthalate Substrate

**DOI:** 10.3390/nano11123313

**Published:** 2021-12-06

**Authors:** Jun Lan, Yong Yang, Song Hu

**Affiliations:** 1 State Key Laboratory of Optical Technologies on Nano-Fabrication and Micro-Engineering, Institute of Optics and Electronics, Chinese Academy of Sciences, Chengdu 610209, China; lanjun19@mails.ucas.ac.cn (J.L.); husong@ioe.ac.cn (S.H.); 2 University of Chinese Academy of Sciences, Beijing 100049, China

**Keywords:** optoelectronic devices, nanostructured polymer film, antireflection coating, finite-difference time-domain method

## Abstract

The application of moth-eye nanostructured polymer film on the flexible polyethylene terephthalate (PET) substrate is an effective way to improve its antireflection (AR) performance. However, many factors affect the AR properties of the moth-eye structure in the actual manufacturing process. Moreover, the antireflection research based on PET substrate has been relatively lacking compared with the silicon substrate. In this paper, we simulate and analyze the AR performance of the moth-eye nanostructured polymer film on PET substrate by using the finite-difference time-domain method within the wavelength range of 400–1100 nm. Simulation results show that the parabola-shaped moth-eye structure (PSMS) can suppress the Fresnel reflection significantly. Moreover, the height and filling ratios are the dominant factors that affect the AR performance of PSMS. Additionally, the base diameter, residual layer thickness, and the refractive index of PSMS polymer film also affect the reflectivity of PET slightly. As a result, an optimal PSMS with base diameter of 400 nm, height of 300 nm, and the hexagonal close-packed arrangement is appropriate, and the solar-weighted reflectivity of PET can be suppressed to 0.21%, which shows a prominent advantage over the bare PET (≈6%). Therefore, this research has promising potential for improving the optical performance of optoelectronic devices by using nanostructured polymer materials.

## 1. Introduction

It is well-known that suppression of optical reflection is essential for many optoelectronic devices [1,2,3,4]. Multilayer antireflection coatings and biomimetic moth-eye structure (MS) are recognized as two effective methods for reducing reflection in the wide wavelength range. However, the application of multilayer antireflection coatings has been accompanied by some problems, such as material selection, thermal stability, thickness mismatch, etc. [5]. Compared with multilayer antireflection coatings, MS can effectively reduce the Fresnel reflection by forming a gradient refractive index (GRIN) profile between the air and the medium of MS [6]. At present, there has been much research carried out to investigate the antireflection (AR) performance of MS based on silicon or glass [7,8,9,10]. However, with the development of technology, optoelectronic devices have gradually transformed to become flexible, ultra-thin, and lightweight. PET is widely employed as device substrate or protection cover in various optoelectronic devices such as solar cells, display panels, transistors, and photodetectors due to its excellent flexibility, light weight, low cost, and high transparency [11,12,13,14]. At normal incidence, the reflectivity is about 6% for a single-side surface of bare PET, which is similar to glass substrates and will degrade the performance of optoelectronic devices [15]. Therefore, in order to guarantee the superior performance of optoelectronic devices, it is meant to reduce the reflectivity of PET by coating the moth-eye structured polymer film. So far, there are few studies of AR based on PET. In general, although there has been some experimental research on the fabrication of AR moth-eye structured polymer film on PET substrate [16,17], there has been very little work on the optimization of preliminary structural design, such as shape, size, material properties, etc. However, it is more complicated, high-cost, and time-consuming to fabricate MS with different geometry sizes for structural optimization. It is worth mentioning that the finite-difference time-domain (FDTD) method has been proved to be able to accurately predict the AR performance of MS by solving the time-dependent Maxwell’s equations [18]. In order to suppress reflectance effectively, it is significant to optimize the shape and key structure parameters of moth-eye structured polymer film by using the FDTD method within the spectral range of 400–1100 nm. In this study, we investigate the influences of both the PET substrate and the thickness of the residual layer on the AR performance of MS. Furthermore, at the end of this paper, we explore the effect of the refractive index of moth-eye nanostructured polymer film on its AR performance.

## 2. Materials and Method

The FDTD method was first developed by Kane Yee in 1966 and is an accurate numerical method to calculate problems in electromagnetics. In this research, all optical simulations were performed using 3D FDTD methods (FDTD solution 2021-v212, Lumerical, Vancouver, British Columbia, Canada). To obtain broadband AR performance for moth-eye structured polymer film, in the simulation, we focused on the solar-weighted reflectance ($R_{SW}$) as the height (*H*), base diameter (*D*), filling ratio ($R_{BDP}$), residual layer thickness, and polymer refractive index variation. It is noted that the $R_{SW}$ considers the influence of solar spectral irradiance, which is defined as the ratio between the reflected photons and the total incident photons and is calculated by using the following equation:

(1) $$R_{SW}=\frac{\int_{400\;\mathrm{nm}}^{1100\;\mathrm{nm}}I_{s}\left(\lambda \right)R\left(\lambda \right)d\lambda }{\int_{400\;\mathrm{nm}}^{1100\;\mathrm{nm}}I_{s}\left(\lambda \right)d\lambda }$$

where $I_{S}\left(\lambda \right)$ is the solar spectral irradiance (i.e., AM 1.5 G) and R(λ) is the total reflectance within the range of wavelength from 400 nm to 1100 nm, respectively.

**Figure 1:**
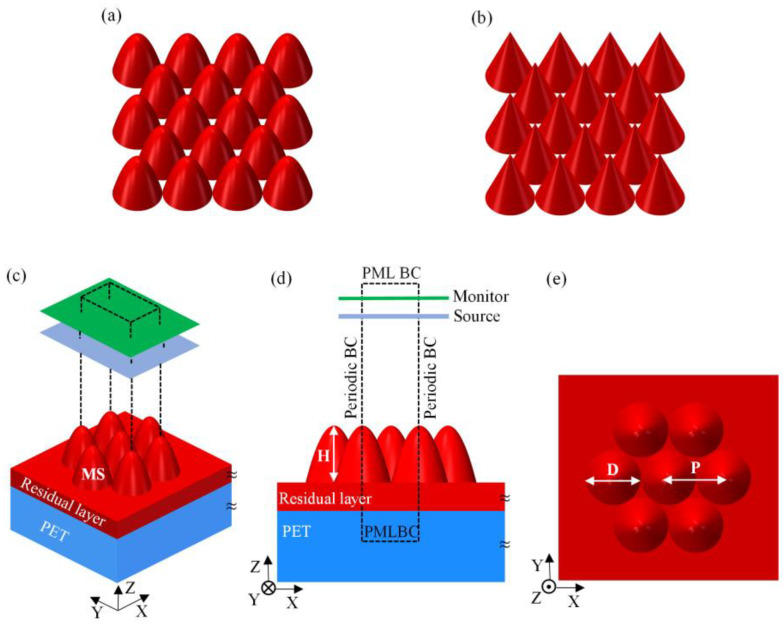
Schematic diagram of 3D physical model and simulation model: (**a**) parabola-shaped moth-eye structure (PSMS) array, (**b**) cone-shaped moth-eye structure (CSMS) array, and (**c**–**e**) parameter setting of simulation unit with the hexagonal close-packed arrangement.

It is well-known that Fresnel reflections can be effectively suppressed by forming a gradual refractive index change between air and materials. Further, both the cone-shaped moth-eye structure (CSMS) and the parabola-shaped moth-eye structure (PSMS) are the most popular AR structures which can suppress the Fresnel reflection effectively [19]. The schematic of structure array models is shown in Figure 1a,b, respectively. Both of them adopted conventional hexagonal arrangement, which is similar to the arrangement of the corneal array structure of natural moth eyes [20,21]. Although many polymers have been used to fabricate antireflection structured polymer film, the UV-curable NOA63 (Norland Products Inc, Cranbury, NJ, USA) stands out for its superior optical properties, appropriate refractive index, environmental adaptability, and suitability for large-area preparation. Specifically, the UV-curable NOA63 has almost no absorption at near-infrared wavelength [22], and its refractive index is close to that of PET. Compared with most polymers, it has a very high Young’s modulus (1.65 GPa) and thermal stability, ensuring that the optoelectronic devices are not vulnerable to the external environment with excellent performance. Moreover, NOA63 polymer films with nanostructures can be rapidly prepared in large areas on PET surfaces by roll-to-roll nanoimprint lithography. It is also worth noting that the high viscosity and fast curing properties of NOA63 ensure antireflection films’ processing speed and durability. Therefore, in this research, the UV-curable NOA63 was used as the structural material of MS, and the influence of different polymers on the AR properties of MS will be analyzed in detail in the last chapter of this paper.

In this simulation, classical and theoretical physical models of PSMS and CSMS are established based on the functional relation of structural parameters as follows [23]:

(2) $$\mathrm{PSMS}:r\left(h\right)=\frac{D}{2}\sqrt{1-\frac{h}{H}}$$

(3) $$\mathrm{CSMS}:r\left(h\right)=\frac{D}{2}(1-\frac{h}{H})$$

where *r*(*h*) is the radius of the cross-section of MS at a height of *h*.

The sketch of the simulation model is shown in Figure 1c–e. Where the dashed frame represents the unit of the simulation region, the optical calculation is considered a one-sided surface of PET. A plane wave light source with normalized intensity and X-axis polarization was normally incident from the air to MS. The perfectly matched layer boundary conditions (PML BC) were set in the direction of propagation of light (Z direction), and the boundary conditions of the X and Y directions were set as periodic boundary conditions (Periodic BC). In the simulation, the refractive index of PET was acquired from the refractive index website [24]. The refractive index of NOA63 polymer was set at 1.56 and the extinction coefficient was not taken into account because it could be ignored [25].

## 3. Result and Discussion

### 3.1. Shape Optimization of the Moth-Eye Structure Array

**Figure 2:**
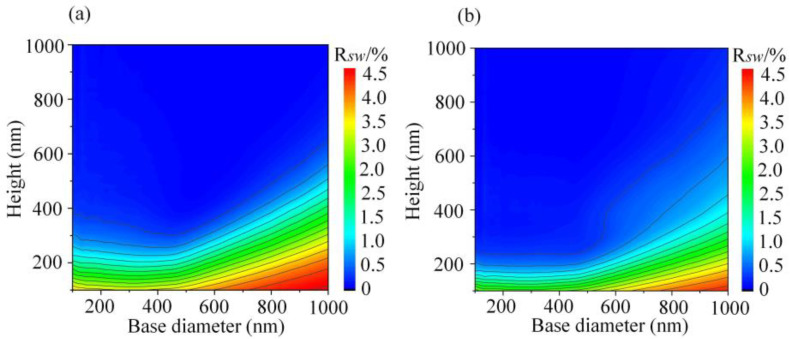
Contour plots of solar-weighted reflectance of (**a**) NOA63 CSMS/PET and (**b**) NOA63 PSMS/PET.

In order to determine the most suitable shape of MS for reducing the reflectivity of PET, the AR properties of CSMS and PSMS were compared. Figure 2a,b show the contour plots of solar-weighted reflectance ($R_{SW}$) of NOA63 CSMS polymer film coated on the PET (i.e., NOA63 CSMS/PET) and NOA63 PSMS/PET in the wavelength range of 400–1100 nm as the diameter and height of MS varied from 100 nm to 1000 nm, where the thickness of the NOA63 polymer residual layer was fixed at 2 μm and MS with the hexagonal close-packed (*D* = *P*) arrangement. 

It is noted that the $R_{SW}$ decreases gradually with the increase in the height when the base diameter is fixed. Further observation of the density of the contour line shows that it mainly becomes sparser and sparser with the increase in the height, which indicates that when MS is high enough, the decreasing rate of reflectivity will be slow. Therefore, there will exist a balance between height and its difficulty of fabrication. On the other hand, when the height of MS is fixed, the $R_{SW}$ shows no obvious changes as the base diameter ranges from 100 nm to 400 nm, which means that the base diameter is not the main factor in affecting the AR properties of subwavelength MS. However, the $R_{SW}$ increases significantly when the base diameter is larger than 400 nm; this phenomenon can be explained by the grating equation under normal incidence lighting [26]:

(4) $$\sin\theta_{d}=m\lambda /n\Lambda ,$$

where *n* is the refractive index of the incident medium, Λ is the grating period, m is the diffraction order, *λ* is the wavelength of the incident light, and $\theta_{d}$ is the diffraction angle. The incident medium is air and $n_{air}=1$. In addition, MS is distributed with the close-packed arrangement, indicating that Λ = *D*. Based on the grating equation, it can be known that when the period of MS is larger than the shortest wavelength of incident light, the high-order diffraction will be generated and cause the diffraction loss [27]. Therefore, in this study, we mainly focus on the design and optimization of MS on submicron scale.

Moreover, it follows from the comparison between Figure 2a,b that PSMS exhibits better AR properties because it can achieve excellent AR performance at a smaller height, which reduces the cost and difficulty of the manufacturing process. Specifically, the effect of shape on AR performance can be explained by using the effective medium theory (EMT). Based on the EMT, subwavelength MS can be regarded as a homogeneous medium with a GRIN profile between air and the NOA63 film. The effective refractive index of subwavelength MS at normal incident can be approximately represented as [23]:

(5) $$n_{\mathrm{eff}}(h)={\left\{f(h)n_{a}^{2/3}+[1-f(h)]n_{b}^{2/3}\right\}}^{3/2}$$

where f(h) is the filling factor, which is equal to the ratio of the cross-sectional area of MS at height of *h* to the area taken up by MS. $n_{a}$ is the refractive index of NOA63 $n_{a}=1.56$, and $n_{b}$ is the refractive index of air ($n_{b}=1$). MS is distributed with the hexagonal close-packed arrangement (*D = P*), and thus the area taken up by MS is equal to $A=\sqrt{3}D^{2}/2$. Further, the cross-section area of MS at height of *h* is:

(6) $$S=\pi r{\left(h\right)}^{2}$$

Therefore, combined with the Equations (2) and (3), the f(h) can be expressed by:

(7) $$f_{PSMS}(h)=\frac{S_{PSMS}}{A}=\frac{\sqrt{3}\Pi }{6}(1-\frac{h}{H})$$

(8) $$f_{CSMS}(h)=\frac{S_{CSMS}}{A}\;\frac{\sqrt{3}\Pi }{6}{\left(1-\frac{h}{H}\right)}^{2}$$

**Figure 3:**
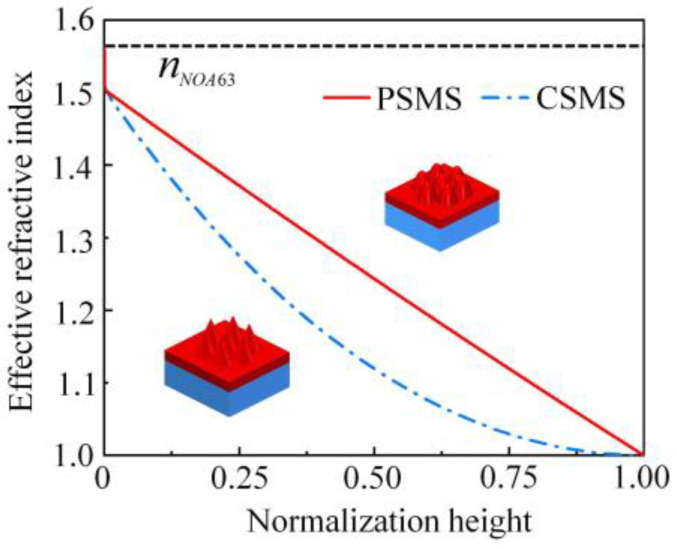
The effective refractive index change between air and the NOA63 film at normal incident. Normalized height 1 represents the peak of the moth-eye structure, and 0 represents the base of the surface of the NOA63 film.

Figure 3 shows the effective refractive index changes between air and the NOA63 film at normal incident. Compared with CSMS, PSMS can provide a nearly linear graded refractive index profile between air and the NOA63 film, which is more efficient to suppress the Fresnel reflection in wide ranges of incident wavelengths [28]. Therefore, the following work is to optimize the parameters of the simulation model of NOA63 PSMS/PET.

### 3.2. Parameter Optimization of NOA63 PSMS/PET

**Figure 4:**
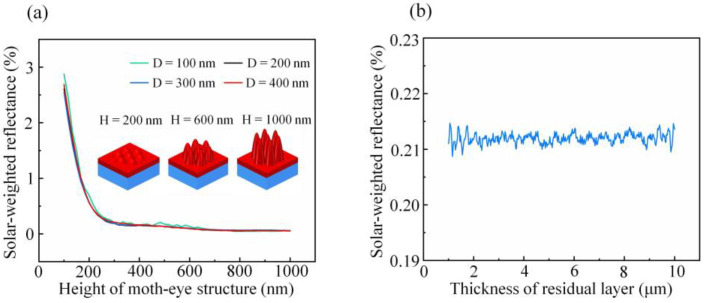
The solar-weighted reflectance for different parameters of (**a**) the height of moth-eye structure and (**b**) the residual layer thickness of NOA63 polymer.

The parameter optimization of the simulation model is helpful to fabricate MS with excellent AR properties efficiently and quickly in practical application. Figure 4a shows the $R_{SW}$ of PSMS with the base diameter *D* varying between 100 nm, 200 nm, 300 nm, and 400 nm. According to the simulation results, the base diameter has little effect on the reflectivity of NOA63 PSMS/PET. As the height of PSMS increased from 100 nm to 300 nm, the $R_{SW}$ values rapidly decreased from around 3% to 0.21%. The reason is that the effective refractive index changes more slowly as height increases, and consequently the Fresnel reflection has been suppressed more efficiently. In addition, when the reflectance is suppressed to a small value, the variation of reflectance with the increase in height is no longer significant. Thus, we can consider that 300 nm is the height with the best benefit for NOA63 PSMS/PET. Moreover, considering the difficulty of MS manufacturing, it is more appropriate to choose subwavelength PSMS with a base diameter of 400 nm.

Further, the effect of the NOA63 polymer residual layer thickness (1–10 μm) on AR performance of NOA63 PSMS/PET is shown in Figure 4b, where the base diameter and height are fixed at 400 nm and 300 nm, respectively, which are also the best parameters as proved above. It can be found that the $R_{SW}$ varies slightly, and the fluctuation range is not more than 0.007% in the residual layer thickness varying from 1 to 10 μm. Therefore, it is almost certain that the residual layer thickness has little influence on the AR performance of NOA63 PSMS/PET under the ideal conditions, which is also consistent with the experimental results of previous relevant articles [29]. This result can be explained by the refractive index of the UV-curable NOA63 being very close to that of PET. Therefore, the Fresnel reflection due to the refractive index difference of the material is effectively suppressed. In addition, the extinction coefficient of the UV-curable NOA63 is negligibly small, so it is not considered in the simulation process. However, in the actual fabrication process, the residual layer thickness of NOA63 film should be as small as possible under the suitable technology. In this simulation, the residual layer thickness is fixed at 2 μm.

**Figure 5:**
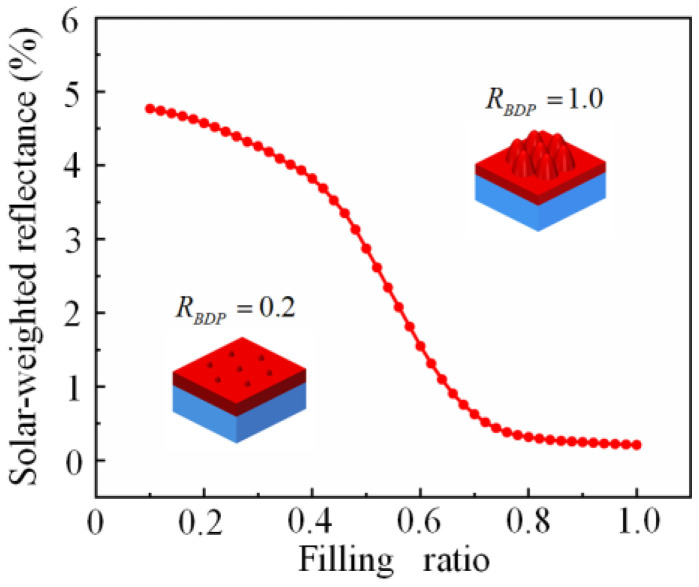
Curve of the solar-weighted reflectance with the filling ratio from 0.1 to 1.

What is worth mentioning is that the filling ratio is considered to be another critical factor besides base diameter and height. For the hexagonal arrangement of the PSMS array, the filling ratio is defined as *D*/*P* (i.e., the ratio of the base diameter of MS to the period between the MS array; $R_{BDP}$). Thus, the filling ratio is directly related to the effective refractive index value of the bottom of the PSMS and ultimately affects the AR performance of the PSMS. Figure 5 shows the $R_{SW}$ of NOA63 PSMS/PET with the filling factor ranging from 0.1 to 1, where the base diameter and the height are fixed at 400 nm and 300 nm, respectively. As shown in Figure 5, the $R_{SW}$ decreases with the filling ratio varying from 0.1 to 1. Specifically, it can be seen that the $R_{SW}$ reduces rapidly with the $R_{BDP}$ varying from 0.1 to 0.7, and then decreases slowly after the $R_{BDP}$ is greater than 0.7. In principle, the $R_{BDP}$ has a significant effect on the reflectivity of NOA63 PSMS/PET. Furthermore, the $R_{BDP}$ should be at least over 0.7, and the best AR properties of PSMS are obtained when the filling ratio is 1. In this simulation, the filling ratio of PSMS with the hexagonal close-packed arrangement is 1.

**Figure 6:**
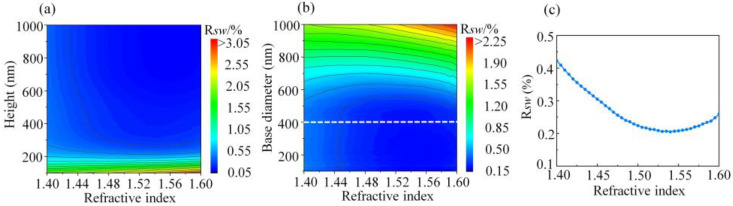
Contour plots of solar-weighted reflectance of PSMS/PET at different polymer refractive indices when (**a**) base diameter is fixed at 400 nm and (**b**) height is fixed at 300 nm. (**c**) The curve of solar-weighted reflectance changing with different polymer refractive indices when *D* = 400 nm and *H* = 300 nm.

In practice, many polymers show the property of reducing the reflectance of PET substrate in experiments. Most of them are low refractive index polymers with refractive index ranging from 1.40 to 1.60. In this study, we further explore the influence of polymer refractive index on the AR performance of PSMS. The $R_{SW}$ of PSMS/PET with different refractive indices of polymer and structural parameters are shown in Figure 6. By analyzing the density and trend of the contour lines in Figure 6a, it can be seen that when the height of PSMS is less than 300 nm, the height is the dominant factor affecting the reflectivity because the $R_{SW}$ changes rapidly with the varying of the height. On the contrary, when the height of PSMS is larger than 300 nm, the contour lines trend changes from horizontal to vertical, which indicates that the refractive index of the polymer becomes the main factor affecting the reflectivity. However, it is noted that the $R_{SW}$ changes slowly with the varying of refractive index. As shown in Figure 6a, the reduction of reflectivity is less than 0.004 (isoline spacing = 0.002) as the refractive index of polymer increases from 1.40 to 1.60. Considering the fabrication difficulty factors, PSMS with a height of 300 nm is appropriate for most polymers to exhibit excellent AR performance.

When the height is fixed at 300 nm, Figure 6b shows the contour plots of the $R_{SW}$ of PSMS/PET as the base diameter increases from 100 nm to 1000 nm and the refractive index of polymer varies from 1.40 to 1.60. It can be seen that when the base diameter is less than 400 nm, the reflectivity is mainly affected by the refractive index of polymer, and the reduction of reflectivity is less than 0.003 (isoline spacing = 0.0015) as the polymer refractive index increases from 1.40 to 1.60. Further analysis shows that the contour line approaches being completely vertical, which indicates that the reflectivity of PSMS is almost not influenced by a base diameter below 400 nm. It is also consistent with the previous simulation result. At the same time, it further proves the correction of selecting the best height with the base diameter of PSMS fixed at 400 nm in our simulation. On the other hand, when the base diameter of PSMS is larger than 400 nm, the $R_{SW}$ of PSMS/PET is mainly affected by the base diameter, and the influence is relatively strong. Based on the above analysis, we can determine that compared with the structural parameters of PSMS, the refractive index of the polymer is not the dominant factor that affects the AR performance of PSMS. In order to further accurately reflect the variation of $R_{SW}$ caused by the polymer refractive index, the curve of solar-weighted reflectance changing with the polymer refractive index when the height is fixed at 300 nm and the base diameter is fixed at 400 nm is shown in Figure 6c. With the increase in the refractive index, the $R_{SW}$ of PSMS/PET decreases at first and then increases. Furthermore, it is more appropriate to choose a polymer with a refractive index around 1.55 to fabricate PSMS for reducing the reflectivity of PET.

## 4. Conclusions

This study investigated the AR effects of moth-eye nanostructured polymer films on top of PET substrate in the wide wavelength range of 400–1100 nm. The shape of the MS array and the design parameters of NOA63 PSMS/PET were optimized by using FDTD simulation. In general, a close-packed hexagonal arrangement ($R_{BDP}=1$) of subwavelength PSMS with height of 300 nm and base diameter of 400 nm exhibits excellent AR performance, which can effectively inhibit the reflectivity to about 0.21%. Furthermore, the base diameter, residual layer thickness, and refractive index of polymers also slightly affect the reflectivity of PET; the determination of these parameters needs to consider the fabrication costs. In principle, the thickness of the residual layer should be as small as possible, and the refractive index of the polymer near 1.55 is more appropriate. This study provides theoretical guidance for the design of broadband antireflection using the moth-eye nanostructured polymer film on PET substrate, which is beneficial to further improving the performance of optoelectronic devices and flexible displays.

## Data Availability

The data is available on reasonable request from the corresponding author.
